# Perspective: divergent mRNA transcription machinery in *Paramecium*

**DOI:** 10.1080/21541264.2025.2570066

**Published:** 2025-10-12

**Authors:** Franziska Drews, Martin Simon

**Affiliations:** Molecular Cell Biology and Microbiology, Faculty of Mathematics and Natural Sciences, University of Wuppertal, Wuppertal, Germany

**Keywords:** Transcription, messenger RNA, PolII complex, *Paramecium*, epigenomics

## Abstract

Proper regulation of transcription involves not only quantitative control of RNA dosage but also ensuring the correct biochemical properties of transcripts. In all eukaryotes, the epigenetic landscape and the dynamic composition of the RNA Polymerase II complex (PolII) interact to control the transcription of translatable mRNA. Decades of research have described dogmatic rules for model organisms, such as the distribution of individual chromatin marks along the transcription unit or the hierarchical phosphorylation pattern in the C-terminal domain (CTD) of the largest PolII subunit RPB1. Besides this canonical mRNA transcription, there are exceptions; on the one hand, not all genes in a species follow the dogma, and on the other hand, there are species that show general divergence from the models, both in the epigenomic landscape and in the genetically encoded PolII. In the recent literature, protists in particular have shifted their attention as they show considerable differences in chromatin structure and PolII complex composition. Here, we aim to enlighten the transcription machinery of the unicellular ciliate *Paramecium* as an exciting model to study a divergent transcriptional machinery for vegetative mRNA and developmental ncRNA transcription.

1.

Transcription of DNA into RNA involves the coordinated interaction of hundreds of proteins. The concept of co-transcriptional modification of messenger RNA (mRNA) by the RNA Polymerase II complex (PolII) incorporates several mechanistic aspects to ensure the quantity and quality of mRNA. In particular, the latter aspect, the realization of the biochemical quality of the produced RNA, breaks with traditional concepts of biology. Proper transcription requires some information in the chromatin. Since many different epigenetic marks along the active gene lead to the dynamic assembly of the PolII complex, they not only regulate the start and stop of transcription and to some extent mRNA quantity, but these histone marks can also regulate RNA modifications by interacting with RNA-modifying enzymes to ensure correct capping, splicing, and polyadenylation.

Epigenomics-, biochemical-, and structural research defined the functional concept of co-transcriptional regulation, which has been established in almost all model organisms. Although most of these studies have involved only yeast and human cells, it was precisely the consistency of the components and mechanisms in these phylogenetically distant species that led to the well-documented concept of canonical transcription of mRNA, which is widely covered in textbooks.

## 1. A canonical transcriptional cycle from yeast to mammals?

Since we want to discuss the characteristics of the ciliate mRNA transcription machinery in this article, we first want to briefly discuss the rather canonical picture that has been obtained through several decades of research, mainly on the yeast and human PolII complex. Transcriptional regulation can be separated into the steps of initiation, promoter-proximal pausing, elongation, and termination, including dynamic re-arrangements of the multi-protein complex during the transcriptional cycle. Transcription is initiated at the promoter through the formation of a pre-initiation complex (PIC) composed of PolII, the Mediator complex, and general transcription factors (GTFs). The PolII complex then frequently pauses after 20–60 nucleotides at the pre-elongation complex (PEC) stage. This promoter-proximal pausing is controlled by Negative Elongation Factor (NELF) and DRB Sensitivity-Inducing Factor (DSIF) (Wu et al. [1]), the latter forming clamps around DNA and nascent RNA (Bernecky et al. [2]; Ehara et al. [3]). The release of the paused polymerase depends on the Positive Transcription Elongation Factor (P-TEFb), which is responsible for differential phosphorylation of several components of the PEC (reviewed Decker [4]). This leads to a replacement of NELF with the PAF complex and an opening of the clamp formed by DSIF through the binding of SPT6 to the complex to form the active elongation complex (EC) (Vos et al. [5]). Promoter-proximal pausing was first described for heat-shock genes in *Drosophila* (Gilmourand Lis [6]; Rougvie and Lis [7]) and then emerged as a critical step in transcriptional regulation in eukaryotes. However, promoter-proximal pausing appears to be a tool to modulate gene expression, rather than an on/off switch, allowing the cell to rapidly activate genes containing paused PolII (Core and Adelman [8]).

PolII activity and dynamic remodeling of the PolII complex are associated with differential phosphorylation of some components. The dynamic phosphorylation in the C-terminal domain (CTD) of the largest PolII subunit RPB1 is well studied. The heptad repeat of tyrosine, serines, prolines and threonines (Tyr ${\;}_{1}$-Ser ${\;}_{2}$-Pro ${\;}_{3}$-Thr ${\;}_{4}$-Ser ${\;}_{5}$-Pro ${\;}_{6}$-Ser ${\;}_{7}$) forms a flexible extension of the catalytic core (Cramer et al. [9]): differential phosphorylation of repeats allows the CTD to dynamically interact with a wide range of nuclear-binding partners, thus regulating the transcription cycle (Buratowski [10,11]; Bentley [12]). The concept of the CTD code means the pattern of phosphorylated serines (2, 5, 7) in the heptad repeats, allowing for a structured modification pattern with a higher level of information for different reader enzymes (Reviewed in Eick and Geyer [13]).

The transcriptional cycle is characterized by a sequential phosphorylation of the PolII CTD. Along the gene body, differential phosphorylation on Ser ${\;}_{5}$ at the 5’-end of genes (involved in capping and promoter proximal pausing) and increased levels of Ser ${\;}_{2}$ phosphorylation at the 3’-end, required for elongation and mRNA end formation, have been described in detail (Buratowski [11]; Heidemann et al. [14]; Bentley [12]). A mass spectrometry study of human and yeast confirmed a regular phosphorylation pattern of most of the repeats along the CTD [15]. Again, in this context, almost all biochemical studies of this important region have been carried out with yeast and mammalian (human) RPB1, showing that the CTD code can be generally applied. For sure, the individual systems show differences, since the number of heptad repeats varies between, e.g., yeast and humans, and analysis of more species shows an increasing number of these repeats with increasing organismal complexity (Eick and Geyer [13]). However, the general system of sequential phosphorylation of Ser ${\;}_{2}$, Ser ${\;}_{5}$, Ser ${\;}_{7}$ is consistent across the species studied. Nevertheless, the CTD code is not the sole “gearstick” for PolII activity, as the linker region between the CTD and the core has been demonstrated to recruit SPT6 to stimulate PolII elongation. Once again, there appears to be significant conservation in this respect, as this is also true for humans (Vos et al. [5]) and yeast (Chun et al. [16]).

The conservation of phosphosites in RPB1 across divergent species, such as humans and yeast, is not the only feature that has led to the idea that this CTD-mediated transcriptional control is canonical. Additional common features include, for instance, that kinases mediating the differential states of phosphorylation show a certain degree of conservation. Several Cyclin-dependent Kinases (CDKs) have been shown to phosphorylate serines within the CTD, with varying specificity, meaning that they do not work mutually exclusively. The CDK7 subunit of TFIIH (KIN28 in *Saccharomyces cerevisiae*) is specific for Ser ${\;}_{5}$ and Ser ${\;}_{7}$, CDK8 (in mammals and yeast) is a Mediator subunit that selectively phosphorylates Ser ${\;}_{5}$ and CDK9 (BUR1 in *S. cerevisiae*) phosphorylates Ser ${\;}_{2}$, Ser ${\;}_{5}$, Ser ${\;}_{7}$ (reviewed in Eick and Geyer [13]). Given the conservation of the CTD code and the kinases, it is not surprising that the pattern of epigenetic marks, such as histone modifications along expressed genes, also shows a high degree of similarity between species. There are promoter-associated sharp peaks of H3K4me4, H3K9ac, and H3K27ac, while H3K36me3 spreads along the gene to facilitate transcriptional elongation, as reviewed in Gates et al. [17].

These various arguments have led to mRNA transcription often being thought of as generally canonical. But is this true in every case? Indeed, exceptions have been observed, both in terms of individual genes in the human genome not following these rules and in terms of some organisms having different genetic and epigenetic requirements for mRNA synthesis. Exceptions which fall into the first category comprise, for instance, high levels of Ser ${\;}_{2}$ phosphorylation detected at the 5’-end of selected human genes (Schwartz et al. [18]). In addition to this deviation from documented rules of PolII, unusual patterns of chromatin marks have also been described in some genes that do not follow the above dogma of distribution along genes. For example, tumor suppressors with exceptionally high transcription levels have broad H3K4me3 domains along the entire gene rather than narrow peaks at the initiation site (Chen et al. [19]). Exactly how this is mechanically linked to the PolII complex remains unknown. Another surprising study challenges the importance of the CTD, showing that human RPB1 protein lacking large portions of the CTD only shows a moderate transcriptional dys-regulation (Yahia et al. [20]). Thus, transcription of individual genes can differ from canonical transcription in the human system. Such deviations are often overlooked in genome-wide analyses or when gene profiles of all genes are plotted and merged.

## 2. What is special about protists, with their transcription machinery, and particularly with ciliates?

The purpose of this article is to draw attention to the divergent PolII complexes of ciliates, which we identified as an exciting example of non-canonical transcription, or maybe more precisely, “divergent” transcription. These unicellular species belong to the protist kingdom – the root of all eukaryotes, since all fungi, plants, and animals have protist ancestors. From an evolutionary point of view, there is a huge knowledge gap, as there are almost no structural and biochemical data on the transcriptional machinery of a protist.

The protist kingdom is the most phylogenetically diverse, and this diversity extends to the RPB1 subunit. Phylogenetic analysis of this component reveals high diversity among protist RPB1 subunits and low identity with RPB1 from non-protist kingdoms (Dacks et al. [21]; Purkayastha and Karmodiya [22]). Although these sequence comparisons concern the entire protein in particular, the analysis of the C-terminal domain of protist RPB1 revealed that many protists lack the classical heptad repeats. Obviously, their absence is not exclusively associated with unicellularity, since the fungi *Schizosaccharomyces pombe* and *Saccharomyces cerevisiae* have these heptad repeats. Within the fungal group, however, it is the aspergillans that do not show exact heptad repeats but very exact serine-proline repeats, while protists such as *Trypanosoma* and *Leishmania* lack any serine-proline periodicity (Eick and Geyer [13]). Recent data show that PolII core components evolved gradually in protists, as homologue identification based on sequence similarity suggests RPB8 and RPB9 to be gained during evolution; RPB4 and RPB12 are absent in many protist classes (Purkayastha and Karmodiya [22]). In contrast, the heptad structure of RPB1’s CTD is thought to have evolved quite early during evolution, being lost and re-invented several times (Yang and Stiller [23]).

To compare and characterize the transcription machinery in our favorite model species, *Paramecium*, we searched for homologues of not only the PolII core complex, but also the pausing and elongation complexes. This provides a comprehensive overview of the canonical components involved in PIC release, pausing release, and elongation in this species. *Paramecium* is a unicellular ciliate that has a long history of being studied in genetic and epigenetic research. A feature that distinguishes ciliate genetics from that of most other species and that makes these organisms interesting for analyzing the transcriptional machinery is the nuclear dimorphism. Generative micronuclei (MIC) and vegetative macronuclei (MAC) reside in a single cell. The MIC is diploid and harbors centromeric chromosomes that divide by classical mitosis. In contrast, the MAC has acentric chromosomes that divide by amitotic division of uncondensed chromatin, which is devoid of heterochromatin (Drews et al. [24]). These unique features of MAC chromosomes raise the question about transcriptional control. Epigenomic analyses have shown the absence of classical repressive histone modifications and a different distribution of nucleosomes and activating histone modifications. The most significant differences from other species are the low nucleosome occupancy in non-expressed genes and the broad, almost gene-covering spread of H3K4me3 in active genes. As this mark typically peaks at the 5’-end of genes and plays a role in PIC assembly, it is evident that the implications of this and other histone marks must be considered throughout the silent transcriptional cycle (Drews et al. [24]).

MIC and MAC differ significantly in terms of transcription. While mRNA transcription occurs in the vegetative MAC, the MIC remains transcriptionally silent during this stage of growth. During meiosis, a ciliate-specific process takes place in which the parental and germline genomes are compared at the RNA level. For more information on this “scanning” process, we refer to the following excellent reviews: Balan et al. [25]; Rzeszutek et al. [26]. It is important to note that during meiosis, two additional transcription events occur to produce genome-wide lncRNAs (i) from the MIC and (ii) from the parental MAC. Some evidence suggests that the composition of the PolII complex differs substantially between meiotic lncRNA transcription and vegetative mRNA transcription. In *Paramecium*, like in all other ciliates, components/genes involved in meiosis and post-meiotic development can be separated from components involved in vegetative processes by their expression pattern determined by a transcriptomics time-course experiment starting from vegetative cells to meiosis to the different steps of developing a new MAC (Arnaiz et al. [27]). This categorization can also be applied to genes encoding transcription factors or PolII-associated components, which have different copies or isoforms that are expressed during vegetative growth or sexual differentiation (Arnaiz et al. [27]).

There is hardly any biochemical data on PolII components in *Paramecium*. An exception is the characterization of the DSIF complex, which has been studied by the Nowak group. They characterized the meiosis-specific components of SPT4 or SPT5 to be necessary exclusively for lncRNA transcription (Gruchota et al. [28]; Owsian et al. [29]). Since the DSIF complex is the only example for which biochemical data on transcription exists, the current dilemma is clear: data on SPT4/5 are only available for meiotic long non-coding RNA transcription. However, there is a lack of epigenomic data, and no analysis has been conducted to determine the importance of pausing-related components in the transcription of lncRNA and mRNA. There is some epigenomic mapping of a few histone modifications from the vegetative MAC that indicate pausing-like behavior of PolII (Drews et al. [30]), but mechanistic analyses of PolII activity are lacking. At this stage, we want to take our first attempt at reverse genetic characterization of the canonical components of the transcriptional machinery in *Paramecium*, to gain an initial understanding of which complexes are conserved and which are not.

## 3. The genetic inventory of the *Paramecium* PolII complex

**Figure 1.**
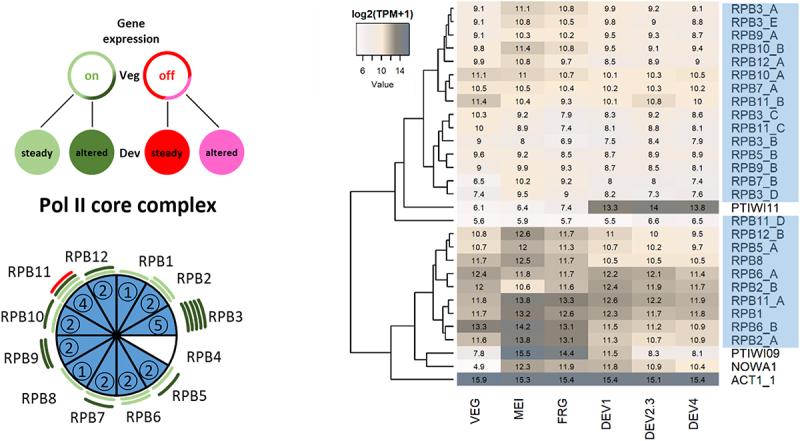
Composition and gene expression pattern of the *Paramecium* PolII core complex. Pie chart illustrates the identified PolII core subunits (blue) with numbers indicating paralogs for each subunit, while white sectors indicate missing orthologs. Genes were categorized into four different groups based on their expression pattern in vegetative growth (Veg) and during the autogamy time course (Dev). The scheme on the left illustrates those four categories, with the color code being transferred to the pie chart. Clustering heatmaps were generated based on gene expression values (log2(TPM +1); indicated by numbers in heatmap) in time course experiments taken from Aury et al. [34], including all identified PolII core complex paralogs and reference genes that show developmentally regulated expression profiles (Arnaiz et al. [27]). For visualization, the heatmap.2 function in R (version 4.5.0) was used (Wickham [35]). See Suppl. Figure S1-S3 for details.

**Figure 2.**
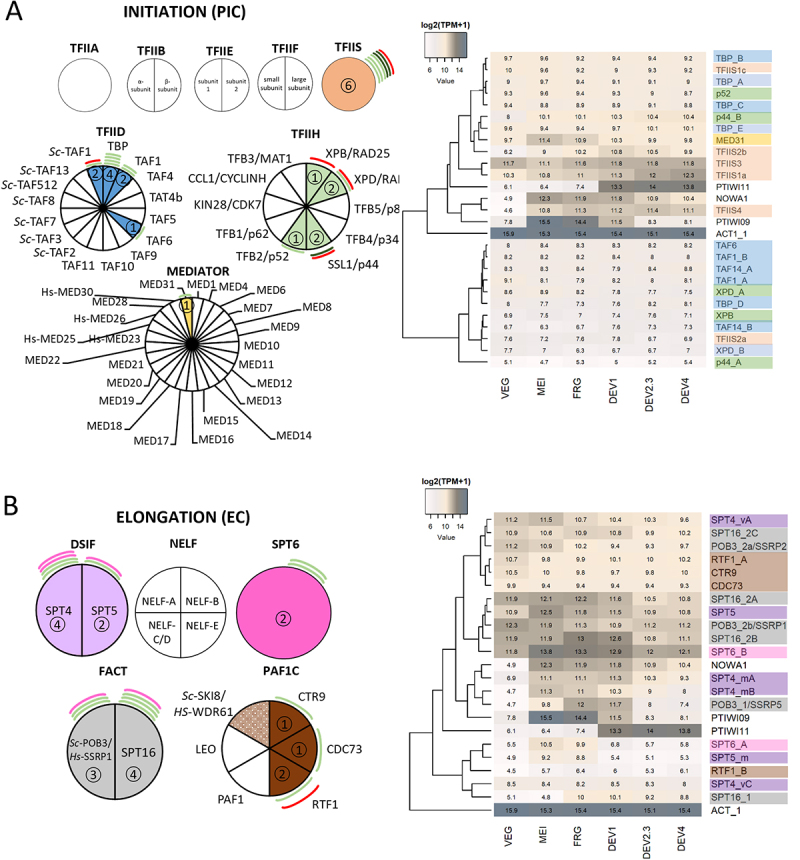
Composition of the *Paramecium* initiation and elongation complex. Identified orthologs and gene expression values (log2(TPM +1)) of the canonical pre-initiation complex (PIC) (A) and elongation complex (EC) (B) are displayed in pie charts and clustering heatmaps comparable to Figure 1. Filler colors of the various charts correspond to the highlighted genes in the heatmaps to the right for readability. Adjacent colored lines correspond to the gene expression patterns during the autogamy time course visualized in the scheme of Figure 1. The dotted section indicates too many hits in the database research.

To identify components of the transcriptional machinery, we used the NCBI Basic Local Alignment Search Tool (BLAST) against the *Paramecium tetraurelia* strain 51 protein sequences (v2.0; https://paramecium.i2bc.paris-saclay.fr/; Arnaiz et al. [31]; Camacho et al. [32]) using amino acid sequences originating from *Homo sapiens* and *Saccharomyces cerevisiae* as queries. Only hits with an E-value below $1e^{-07}$ were considered for our analysis. In Figures 1 and 2, we summarize the expression profiles of the corresponding genes of the PolII core, PIC, and EC and their paralogs during vegetative growth and the sexual process of autogamy based on the RNA-seq data provided by Arnaiz et al. [27].

### 3.1. The *Paramecium* PolII core complex

The PolII core machinery consists of 12 subunits that show a high degree of conservation among yeast and humans, which we all identify by database search, except for one of the smallest subunits: RPB4 (Cramer et al. [33]). Interestingly, only RPB1 and RPB8 are single-copy genes, while all other subunits are encoded by at least two genes, mostly paralogs from whole-genome duplications, of which the *Paramecium aurelia* complex underwent three (Aury et al. [34]).

It becomes obvious that expression profiles of the paralogs are different at least for some of the pairs (Figure 1): in several instances, one paralog is continuously expressed during vegetative growth, whereas the duplicate(s) is developmentally expressed. This indicates a sub-functionalization of those duplicated genes into mRNA expression during vegetative growth and lncRNA transcription during development. The profile of silent to up-regulated in development (pink) is not observed for any subunit of the core complex; all components show a steady state expression level at all time points. The possession of multiple gene copies, being variously expressed, provides at least the genetic requirements to generate life-cycle-dependent complexes of varying composition and modified function. This observation can be readily extrapolated to the number of identified PolII core complex paralogous genes for RPB3 and RPB11, showing the highest numbers of paralogs with vegetative and developmentally regulated expression profiles. RPB3 and RPB11 subunits form a heterodimer, which can be seen as a platform for PolII assembly, thus setting the core of the transcription machinery, as their less conserved domains are responsible for interaction with other PolII core components (Cramer et al. [33]). The possession of multiple variants of the initial PolII platform subunits provides a molecular basis for the assembly of divergent lifecycle-dependent PolII core machinery in *Paramecium*.

### 3.2. The *Paramecium* pre-initiation complex

As introduced, transcription initiation starts by binding of activators upstream of the promoter and transcription start site (TSS), followed by the recruitment of co-activators like the Mediator complex and chromatin remodelers, which promote attachment of GTFs such as TFIID, TFIIA, TFIIF, TFIIE, and TFIIB that guide PolII to its binding site, and it is estimated that the GTFs and the Mediator complex are composed of about 60 proteins in yeast (Kornberg [36]). Upon PIC formation, RNA synthesis is initiated once 10-15bp of the DNA is helix unwound, allowing PolII to pass through (Li et al. [37]).

Transcription factor II D (TFIID) is the first factor to assemble at the promoter sequence through interaction with the TATA-binding protein (TBP), of which we were able to identify four variants by BLAST search (Figure 2(A)). Moreover, the largest subunit TAF1 (TBP-associated factor 1), which acts as a landing platform and is crucial for TFIID assembly (Bernardini et al. [38]), is identified and shows expression during vegetative growth. Strikingly, GTFs like TFIIA, TFIIB, TFIIE, and TFIIF, supporting interaction of the PolII core complex, TFIID, and the DNA, cannot be detected by sequence similarity search, as visualized in Figure 2(A).

One of the main complexes for the transcriptional activation of PolII is the Mediator complex, composed of 26 core components in humans, which interacts with the RPB1 CTD and guides chromatin dynamics and PolII phosphorylation to regulate gene expression (Luyties and Taatjes [39]). *Paramecium* lacks these components – only the highly conserved MED31 subunit can be identified. Another ciliate, *Tetrahymena*, also lacks most of the Mediator components as well, while mass spectrometry analyses revealed a divergent complex of MED31 and ciliate-specific components (MED31-interacting *Tetrahymena*-specific proteins (MITS 1–13) (Garg et al. [40]). The functional analysis of this Mediator complex in *Tetrahymena* demonstrated its involvement in regulating developmentally specific genes and developmental non-coding RNA transcription, suggesting that it has the capacity to regulate PolII transcriptional activity. ChIP-seq of Med31 revealed a distribution along whole open reading frames, with highly and lowly expressed genes being covered. Thus, the Mediator in *Tetrahymena* is not exclusively linked to positive transcription regulation but also negatively regulates at least a subset of genes, showing again not only divergence in composition but also in function (Garg et al. [40]). Another study additionally identified three Mediator-associated factors in *Tetrahymena* (Tian et al. [41]), EMIT1, EMIT2, and RIB1, none of which we can identify by our BLAST search (not included in Figure 2). Moreover, we are not able to identify the *Tetrahymena*-specific proteins MTIS1-13 either, which is why the composition of the Mediator complex in *Paramecium* remains elusive. A comparative genomics study by Bourbon [42] predicted multiple Mediator subunits in *Tetrahymena* by a sequence motive analysis and structure modeling, leading to the idea of Mediator being somewhat conserved in the main module structure and including the highly conserved Med31 subunit. So we conclude that the Mediator shows a high degree of divergence even among the ciliate clade with various functions in positive and negative regulation of transcription.

PIC assembly is further guided by TFIIB, a GTF interacting with the TBP for the recruitment of PolII. While we did not identify a TFIIB yeast or human ortholog in *Paramecium*, Cai et al. [43] described two TFIIB-like proteins, EMIT3 and TFIIBL1, to be involved in ncRNA transcription from TE-related sequences in *Tetrahymena* or being expressed in vegetative growth, respectively. We have been able to identify a potential EMIT3 ortholog, showing conserved amino acid residues and vegetative expression profile, which might be a promising candidate for functional analysis of the TFIIB in *Paramecium*.

The *Paramecium* TFIIH, being described as a 10-subunit complex in yeast and mammals relevant for DNA unwinding and CDK7 recruitment for PolII phosphorylation, is probably also very divergent. In the *Paramecium* genome, we can only identify clear orthologs to XPD and XBP, being relevant for CDK recruitment, and their interaction partners p44 and p52, which have also been identified in *Tetrahymena* recently (Egly and Coin [44]; Greber et al. [45]; Cai et al. [43]), with XPB being expressed only at low levels. All other TFIIH subunits do not show orthologs in the *Paramecium* genome. XPD and XPB subunits have multiple functions: promoter opening and transcription initiation, CDK recruitment for PolII CTD phosphorylation, as well as a function in nucleotide excision repair (reviewed in Rimel and Taatjes [46]). While XPD is known to link the core and CAK modules in yeast and humans, XPD helicase activity appears to be dispensable for transcription, though it is necessary for DNA repair. This suggests that XPD acts as a structural component in transcription. Moreover, TFIIH without XPB can still initiate transcription (Kuper et al. [47]; Winkler et al. [48]). Similarly, XPB-depleted TFIIH can still be recruited to promoters and initiate transcription (Alekseev et al. [49]). Consequently, while the identification of these TFIIH components was achieved, further investigation is necessary to ascertain their role in transcription initiation and PolII guidance rather than in DNA damage repair.

Interestingly, Mediator interacts with TFIIH and stimulates CDK activity. How this interaction is achieved in *Paramecium*, with multiple TFIIIH and Mediator subunits being absent, remains elusive.

As visualized in Figure 2, most of the canonical GTFs have not been identified by sequence similarity search. One exception is TFIIS, one of the first GTFs being described in regulating the shift from initiation to elongation, which is identified and studied in *Paramecium*. TFIIS helps to cleave nascent transcripts from backtracked PolII, a state where the transcript is mislocated in the PolII complex and needs to be cleaved. Thus, TFIIS promotes elongation. The factor is encoded by six paralogs in *Paramecium*, which show different expression patterns in sexual development and have been studied in terms of ncRNA production in MIC meiosis (Maliszewska-Olejniczak et al. [50]), but the vegetatively expressed forms are not yet studied in terms of mRNA production.

### 3.3. The *Paramecium* elongation complex

PolII proceeds to the elongation stage by losing contact with general transcription factors at the promoter. In metazoans, PolII pauses 0–100 bp downstream of the (TSS), a process introduced by NELF and DSIF, the latter comprising SPT4 and SPT5, with SPT5 being conserved across all kingdoms (Guo et al. [51]). NELF and DSIF, the latter interacting with nascent RNA, DNA, and PolII, hinder the incorporation of nucleotides thereby blocking PolII in effective elongation. To release PolII from promoter-proximal pausing, NELF is phosphorylated by the positive elongation factor B (P-TEFb) (Decker [4]), resulting in NELF dissociation and by phosphorylation of DSIF, the factor switches into a positive elongation factor for PolII release. Additional phosphorylation recruits elongation factors associated with the active gene body and chromatin modifiers such as the Polymerase-associated factor 1 complex (PAF1) and histone chaperone SPT6 for promoting elongation.

Our sequence similarity analysis confirms that NELF misses orthologs, but SPT4 and SPT5 are conserved in *Paramecium* (Figure 2(B)). Developmental SPT4 and SPT5 isoforms have been studied and appear to be involved in ncRNA transcription during meiosis (Gruchota et al. [28]; Owsian et al. [29]). The FAcilitates Chromatin Transcription (FACT) complex promotes elongation by interacting with histones and acting in concert with P-TEFb to counteract the negative elongation activities of DSIF and NELF, thus linking the process of initiation and elongation of PolII (Mason and Struhl [52]). We identified both subunits of FACT in the *Paramecium* genome, which are vegetatively expressed or developmentally regulated (Figure 2(B)). One paralog, SPT16-1 has already been studied in development and acts as a chromatin remodeler promoting DNA excision (de Vanssay et al. [53]).

Transcription elongation and 3’-end processing are further controlled by the PAF1 complex. For PAF1, only three out of five subunits, RTF1, CTR9, and CDC73, can be identified in *Paramecium* with SKI8/WDR61 (Chen et al. [54]), giving too many hits in our BLAST search to make a reasonable decision on the number of orthologs/paralogs. Elongation is guided by DSIF remaining on the PolII complex, acting as a platform for recruitment of elongation factors such as RTF1 (Wier et al. [55]). Structural analysis of human PolII shows that the PAF complex replaces NELF, and SPT6 binds to the complex to open the clamp by DSIF to form the fully active EC (Vos et al. [5]). SPT6, which acts as a histone chaperone and is encoded by multiple copies in *Paramecium* (Figure 2(B)), guides partial nucleosome unwrapping along the gene body while the PAF1 complex functions as a positive allosteric regulator of elongation. It recruits histone modifiers promoting transcription by deposition of H3K4me3 and H3K36me3 toward the 3’-end of the gene body.

### 3.4. Summary: differential expression patterns of PolII complexes during development

**Figure 3.**
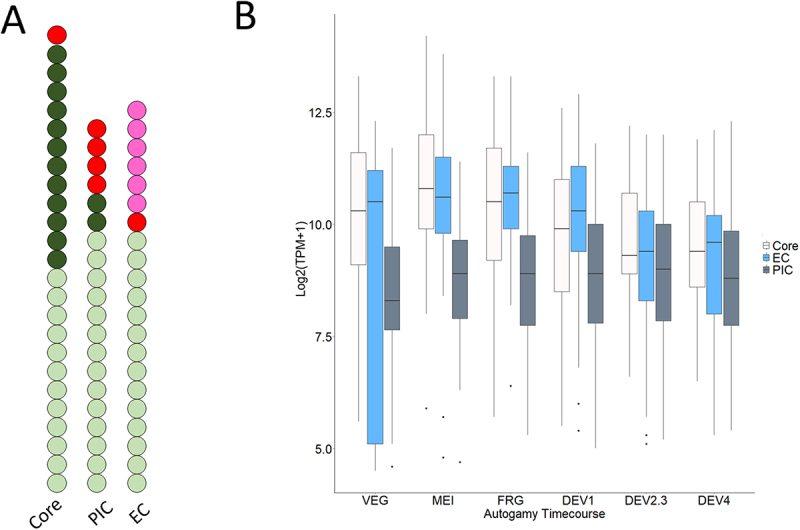
Gene expression dynamics of the PolII transcription machinery in *Paramecium*. A. Summary of gene expression groups for PolII core, PIC, and EC components. Each bubble reflects one identified subunit, including paralogs, while the color code corresponds to the scheme in Figure 1. B. Boxplot of gene expression values (log2(TPM +1)) in autogamy time course with grouping of all identified genes in this study to the PolII core, PIC, or EC, respectively.

Figure 3(A) summarizes the observed expression patterns of all identified proteins of the PolII core, PIC, or EC. As the color code indicates, the EC seems to be composed of subunits with more variable expression profiles. Some subunits appear to be especially active only in development (pink), while most components of the PolII core and PIC have a steady-state expression level in vegetative growth and development (green). Thus, one can speculate that *Paramecium* regulates the shift from mRNA production to ncRNA synthesis in development on the level of elongation. Figure 3(B) visualizes expression values of all subunits: while components of the PIC show a narrow, steady expression during the life cycle, core components are expressed at higher levels, while EC show a more dynamic expression pattern.

## 4. What could be the role of serin/threonine phosphorylation in paramecium?

When it comes to studies on yeast and human PolII phosphorylation, the CTD of RPB1 is the first thing that comes to mind. In CTD, regular heptad repeats (Tyr ${\;}_{1}$-Ser ${\;}_{2}$-Thr ${\;}_{4}$-Ser ${\;}_{5}$-Ser ${\;}_{7}$) were described, which allow for a structured phosphorylation pattern. The CTD code means the pattern of phosphorylated serines (Ser ${\;}_{2}$, Ser ${\;}_{5}$, Ser ${\;}_{7}$) in the heptad repeats, allowing for a structured modification pattern with a higher level of information for different reader enzymes (Buratowski [10,11]; Bentley [12]). A mass spectrometry analyses confirmed a regular phosphorylation pattern of most of the repeats along the CTD [15], so the regular pattern seems to be important for the function. Next to serines, also Thr ${\;}_{4}$ was described as a kinase target in association with transcriptional elongation (Hintermair et al. [56]).

**Figure 4.**
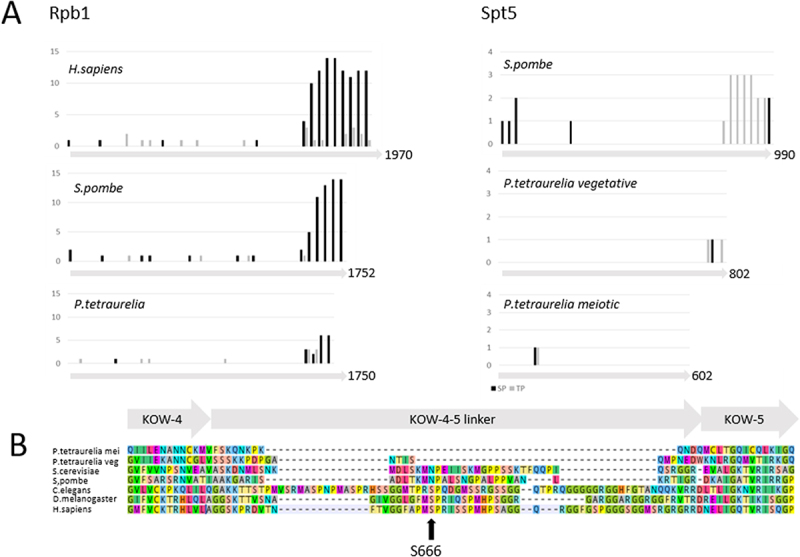
Phosphorylation sites in RPB1 and SPT5. A. Number of TP (gray) and SP (black) dipeptides in RPB1 orthologs (50AA bins) and SPT5 orthologs (25AA bins). Grey arrows indicate the length of proteins in number of amino acids at the respective C-terminal end. B. ClustalX alignment of SPT5 orthologs (*Paramecium*, yeast, human, *Caenorhabditis elegans*, *Drosophila melanogaster*) with zoom in the KOW4-5 linker region harboring the serine 666 phosphorylation site.

While the number of heptad repeats is lower in *S. pombe* compared to human CTD, they are clearly present there. *Paramecium* RPB1 does not reveal any repeat structure nor any motif. Phosphorylation in a higher-order system that is part of a higher-level information system, such as the CTD code, is impossible here. However, the C-terminal area of RPB1 is enriched for SP and TP dipeptides (Figure 4A), and this suggests that differential phosphorylation could occur there. Since the classical heptad repeats do not exist in *Paramecium* RPB1, it is also not surprising that we cannot identify any CDK7/8/9 orthologs in *Paramecium*: since a Blast search indeed retrieved many results of kinases (using *H. sapiens*, *S. cerevisiae* and *S. pombe* as queries), mostly cyclins, phylogenetic comparison does not allow us to identify clear orthologs, while human and fungi CDKs nicely cluster (Suppl. Figure S4-S5).

What could the regulatory role of *Paramecium* RPB1-CTD be? Could it be comparable to the region that links the CTD to the catalytic core? This linker becomes differentially phosphorylated, binds to SPT6, and causes pause release of PolII elongation. This mechanism appears to be conserved: it has been described in human RPB1 by P-TEFb phosphorylation (Vos et al. [5]), and in yeast by BUR1/CDK9 phosphorylation (Chun et al. [16]). It is tempting to speculate that the *Paramecium* CTD could have a function similar to the linker, in particular in pausing release: This mechanism indeed needs to be divergent since NELF could not be identified by sequence similarity search (Figure 2B). One should also consider the *Paramecium* RPB1 CTD involved in phase separation. PolII condensates *in vivo* depend on the length of the CTD and the degree of phosphorylation (Boehning et al. [57]). This property has been observed in mammals and yeast, but it is not known whether the ability of PolII to form condensates is also conserved in a wider range of species. Would this make sense in *Paramecium*? We can only speculate about the factors of a huge nucleus [30 µm], 800n polyploidy, and an inability to condense chromosomes. Speckle formation of active PolII seems reasonable, but has never been demonstrated. Many more biochemical data from humans allow for the dissection between different condensates: promoter condensates to concentrate proteins such as transcription factors at the initiation sites of highly transcribed genes, and condensates of phosphorylated CTD at gene bodies (splicing speckles or paraspeckles) (Cramer [58]).

Next to RPB1, also the C-terminal domain of SPT5, the most conserved component, shows differential phosphorylation exclusively mediated by CDK9 (Yamada et al. [59]). In metazoans, SPT5 has two major phosphorylation sites: serine 666 in the KOW4/KOW5 linker and individual threonine residues in the repeats (reviewed in Song and Chen [60]), which can vary between species. In *S. pombe* for instance, the nonapeptide TPAWNSGSK becomes phosphorylated at threonine 1 (Pei and Shuman [61]). The comparison of *Paramecium* SPT5 isoforms to other species reveals the absence of any repeat structure in the C-terminal region. Although in the vegetatively expressed isoform some SP and TP dipeptides are present in the C-terminal area, these are completely missing in the isoform which was previously shown to be involved in lncRNA transcription during meiosis (Owsian et al. [29]). Future research will need to clarify whether some phosphorylation occurs in the vegetative isoform and, if so, whether this is related to pausing release. Assuming that global, chromosome-wide lncRNA transcription during meiosis does not involve RNA capping or highly sophisticated PolII rearrangements in terms of pausing and elongation complexes, one could assume that differential phosphorylation is not required in meiotic SPT5. Sequence comparisons of SPT5 demonstrate that pausing release is regulated differently in *Paramecium* by SPT5. This is also confirmed by the absence of serine 666 (Figure 4B).

## 6. Conclusion

The PolII complex and MAC chromatin of *Paramecium* show significant differences compared to yeast and mammals, for which most biochemical studies have described conserved, albeit not identical, transcription mechanisms. In this article, (Drews et al. [24]) we aim to demonstrate that the transcriptional machinery of ciliates is not only of interest due to its obscurity but also has the potential to shed light on non-canonical processes in other species, including humans.

Several biological special features distinguish the MAC from any other nucleus: it divides amitotically, so there is no need for condensation, and heterochromatin is generally absent (Drews et al.). An aspect that is easily overlooked is that these amitotic divisions do not require transcriptional shutdown during cell division, which may be a mechanism employed by unicellular organisms to increase division rates. The highly concerted removal and re-import of PolII, which also involves CDK9-mediated release of paused PolII (Liang et al. [62]), is not necessary in the MAC, and this may contribute to the distinct requirement for differential phosphorylation. However, the question arises as to whether these special characteristics of the MAC also occur in other biological contexts. It is, of course, provocative to compare ciliates with cancer, but polyploid giant cancer cells (PGCCs) at least exhibit a similar problem of polyploidy and amitosis (Mirzayans and Murray [63]). This example should at least emphasize once again that it is worthwhile to expand the image of the canonical cell nucleus and canonical transcription to include these exceptions to understand the entire biology of transcription.

Our database analysis of PolII-associated factors revealed that the basal transcription factors are either absent, based on sequence similarity search, or highly divergent. This means that they cannot be identified by reverse genetics based on homology. This makes sense when you consider that intergenic regions harboring putative regulatory elements in the *Paramecium* genome are extremely small and are sometimes simply absent (Aury et al. [34]). Furthermore, the only epigenomic analysis of the MAC also suggested that more regulatory capacity for transcription is moved into the gene body (Drews et al. [30]). This brief overview of conserved and non-conserved/less conserved PolII components aims to stimulate interest in ciliate transcription, which is an excellent model for two key aspects: 1) PolII re-organization between ncRNA/mRNA transcription, and 2) NELF-independent pausing.

The first aspect, the spatial and temporal dissection of PolII activities during meiotic ncRNA synthesis and vegetative mRNA transcription, seems to be quite specific to ciliates. However, the general problem of how to reorganize a complex to create products of different quality is the same, even in a single nucleus. Our preliminary analysis suggests that there are a considerable number of meiosis-specific isoforms, particularly concerning transcriptional elongation (Figure 3). Studying PolII elongation will be challenging, even in vegetative mRNA synthesis, since almost nothing is known about the components or elongation-associated histone modifications (e.g., H3K36me3). However, meiotic ncRNA transcription involves chromosome-wide transcription, so the need for efficient elongation is more evident than in the MAC, where there are tiny transcriptional units due to extremely short introns. The efficient transcription of entire MIC chromosomes into ncRNA is particularly important, as it distinguishes *Paramecium* from *Tetrahymena*, thus explaining the aforementioned differences in the Mediator complex. In *Tetrahymena*, only parts of the chromosomes are transcribed into ncRNA, e.g., those that are to be eliminated (Schoeberl et al. [64]). Therefore, some promoter-like sequences are required for initiation, and presumably a different elongation system for this kind of local ncRNA transcription.

The second aspect is NELF-independent pausing. NELF is absent in ciliates and many other species, e.g., such as *S. pombe*. A recent study also described a slowdown of PolII after the transcript start in these NELF negative species: since the pausing position is not that discrete compared to classical mammalian pausing, the authors speak about “proto-pausing” and hypothesize about chromatin factors slowing down PolII until P-TEFb recruitment (Chivu et al. [65]). The proto-pausing pattern is quite similar to what we have found by PolII ChIP-seq in *Paramecium*, which appears to become a fascinating model to study these mechanisms of transcriptional control, as it represents one of the rare model species combining the features of NELF independent pausing with the absence of heptad repeats. Furthermore, a mechanistic analysis of how, where, and when phosphorylation occurs, which kinases are recruited and by whom, and the epigenomic background of the genes will certainly reveal new and exciting aspects of transcriptional control.

Will these findings only apply to *Paramecium*? A basic understanding of how PolII can catalyze mRNA synthesis beyond the canonical description will also provide insight into the role of genes that do not follow the canonical mechanisms. As we explained above, even in the human system, not all genes are transcribed according to the standard rules. There are also other signatures in the epigenome and CTD phosphorylation. It is precisely these examples and variations of the canonical narrative that are important for analyzing and understanding the dynamics of the PolII complex, both genome-wide and gene-specific, in humans. Analyzing ciliate PolII complexes will certainly contribute to our understanding of the basic principles underlying both mRNA transcription and the dynamic switch between mRNA and ncRNA transcripts.

In addition to the bilateral comparisons with the human system, we would like to emphasize the evolutionary aspects of the outstandingly interesting aspects of ciliate transcription machinery. A deeper understanding of ciliate mRNA synthesis and PolII dynamics concerning ncRNA and mRNA transcription would offer valuable insights into the evolution of the transcription machinery. This is particularly pertinent given that protists are believed to be the origin of all multicellular organisms.

## Supplementary Material

Supplemental Material
