# Supplementary material for: Perspective: divergent mRNA transcription machinery in Paramecium
Source: Transcription. 2025 Oct 12;16(4-5):398–412. doi: 10.1080/21541264.2025.2570066 (PMC12716044; doi:10.1080/21541264.2025.2570066)
Supplement: Supplemental Material [file KTRN_A_2570066_SM1573.pdf]

Supplementary Material

1 FIGURES

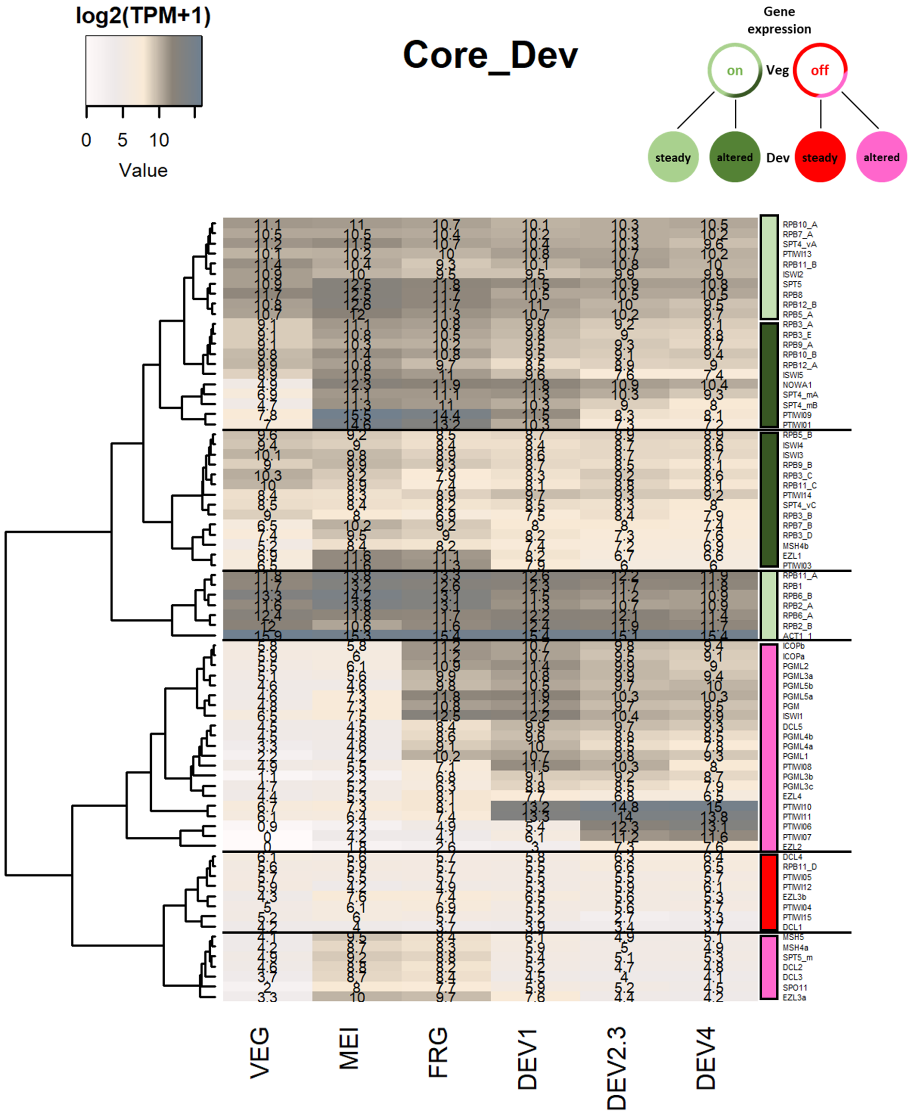

Figure S1. Gene expression patterns of PolII core complex . The scheme illustrates four gene expression categories, with the color code being transferred to the clustering heatmap below. Heatmaps were generated based on gene expression values (log2(TPM+1)) in time course experiments taken from Aury et. al (2006), including all identified PolII core complex genes and paralogs (*n=25*). Reference genes that have been described as being differentially regulated during autogamy (*n=53*) are included, and clustering patterns of these genes were used as a basis for sorting PolII core complex genes into expression groups according to the scheme.

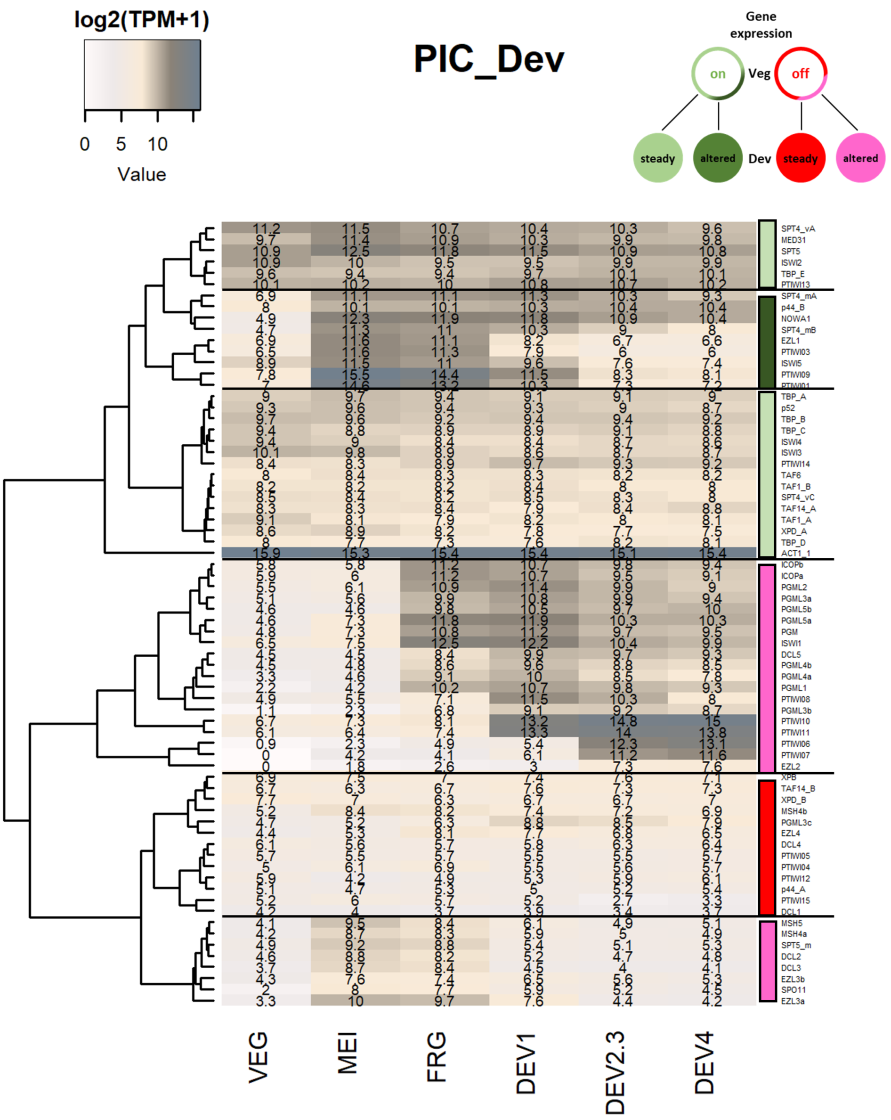

Figure S2. Gene expression patterns of PIC components. The scheme illustrates four gene expression categories, with the color code being transferred to the clustering heatmap below. Heatmaps were generated based on gene expression values (log2(TPM+1)) in time course experiments taken from Aury et. al (2006), including all identified PIC subunits encoding genes and paralogs (*n=23*). Reference genes that have been described as being differentially regulated during autogamy (*n=53*) are included, and clustering patterns of these genes were used as a basis for sorting PIC genes into expression groups according to the scheme.

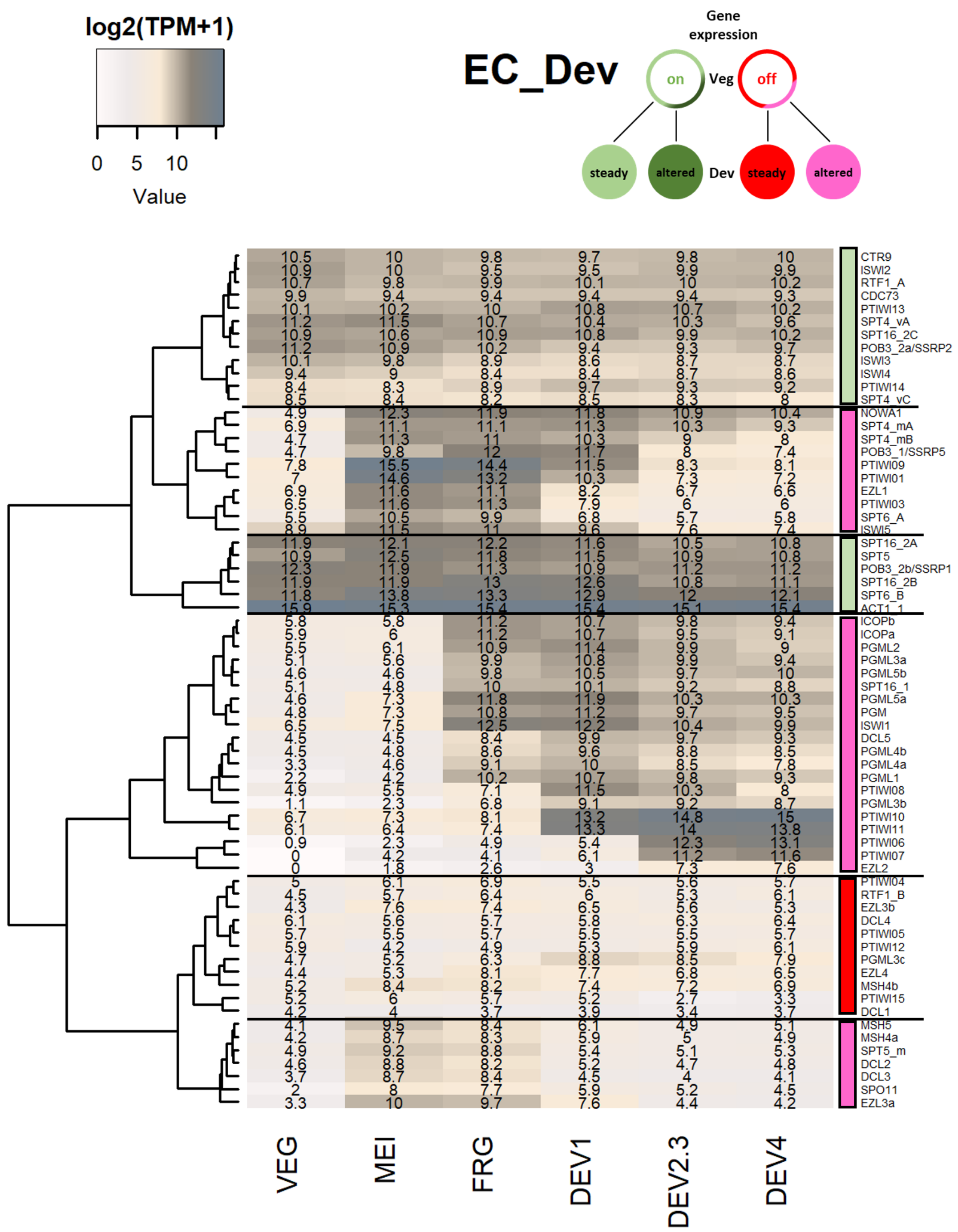

Figure S3. Gene expression patterns of EC components . The scheme illustrates four gene expression categories, with the color code being transferred to the clustering heatmap below. Heatmaps were generated based on gene expression values (log2(TPM+1)) in time course experiments taken from Aury et. al (2006), including all identified EC subunits encoding genes and paralogs (*n=19*). Reference genes that have been described as being differentially regulated during autogamy (*n=53*) are included, and clustering patterns of these genes were used as a basis for sorting EC genes into expression groups according to the scheme.

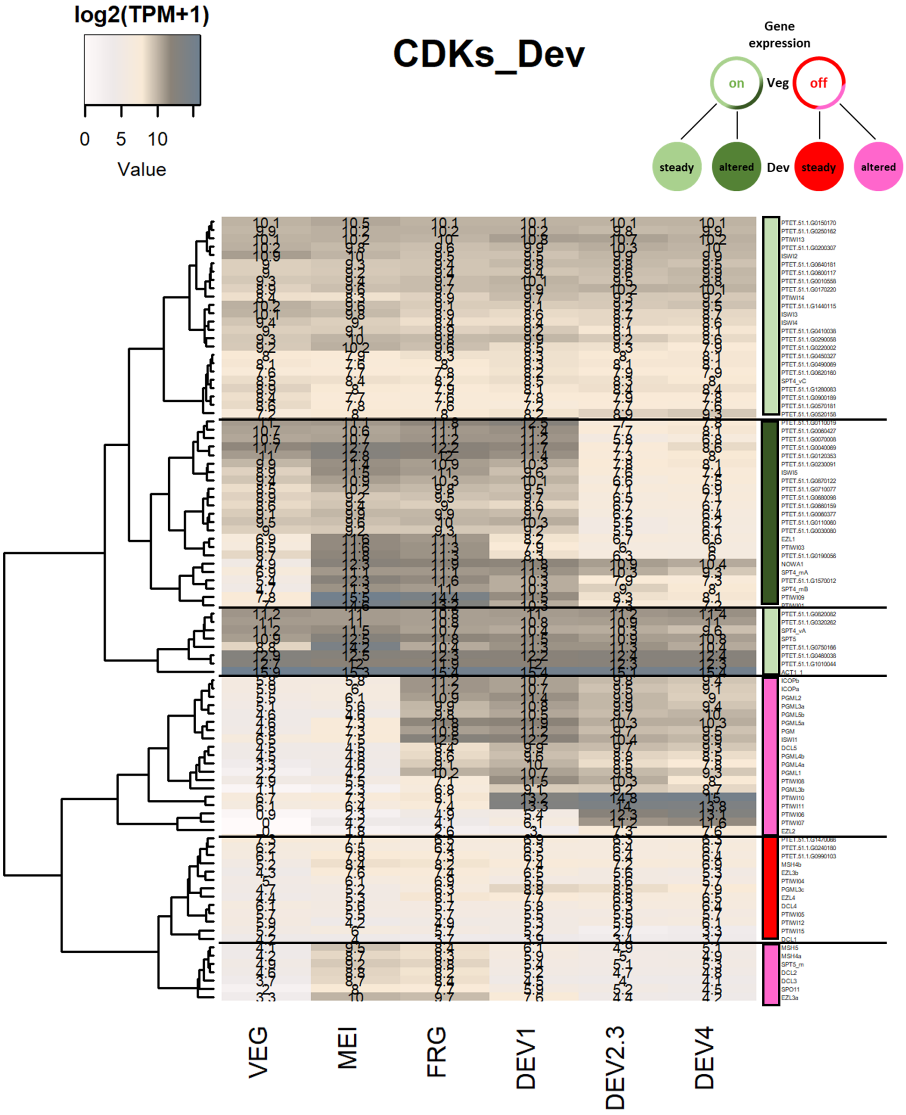

Figure S4. Gene expression patterns of putative CDKs. The scheme illustrates four gene expression categories, with the color code being transferred to the clustering heatmap below. Heatmaps were generated based on gene expression values (log2(TPM+1)) in time course experiments taken from Aury et. al (2006), including all putative CDK encoding genes and paralogs (*n=41*). Reference genes that have been described as being differentially regulated during autogamy (*n=53*) are included, and clustering patterns of these genes were used as a basis for sorting CDKs genes into expression groups according to the scheme.

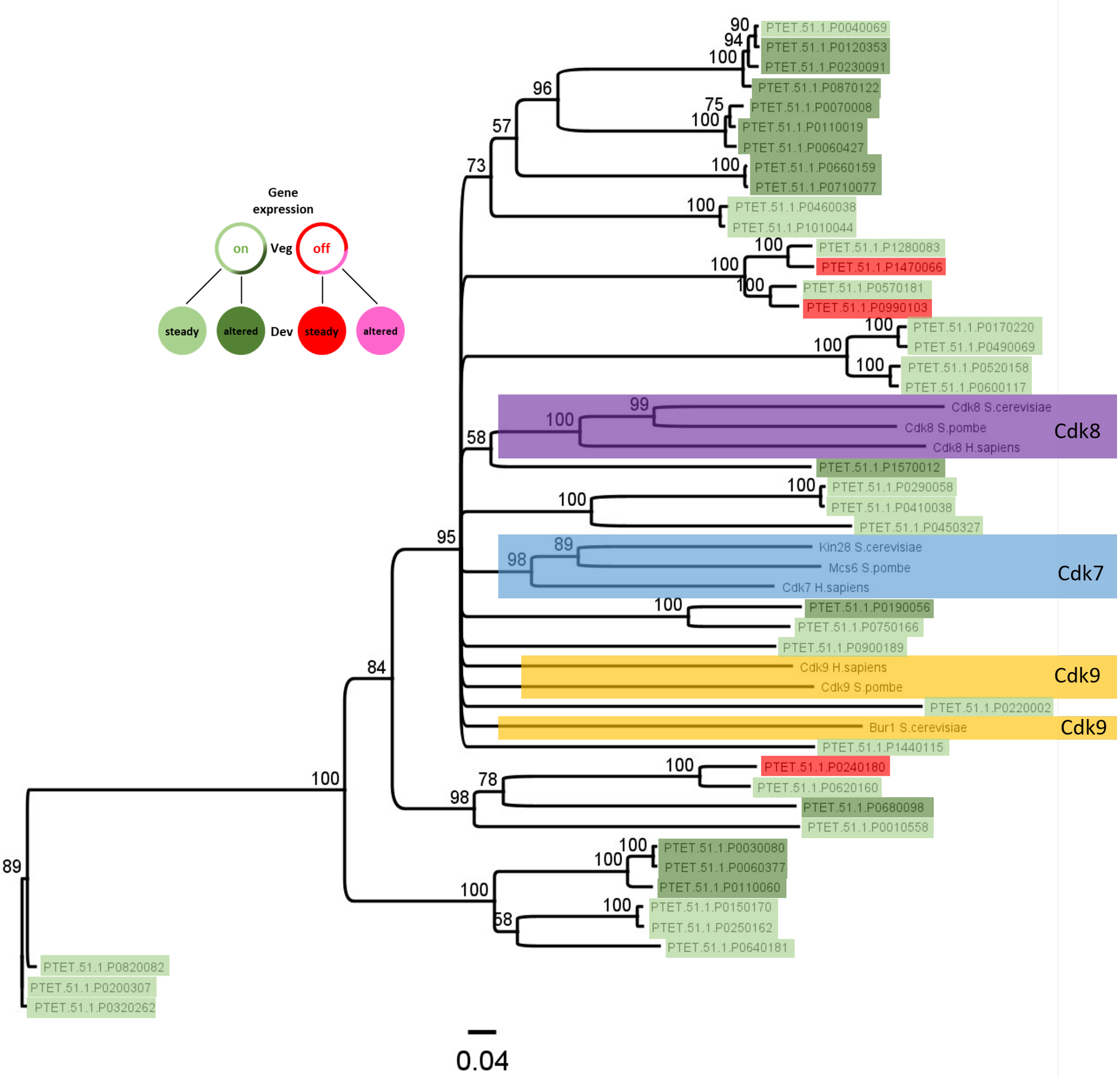

Figure S5. Phylogenetic tree of putative CDKs. Results were retrieved by BLAST screen for orthologs of human, *S. cerevisiae* and *Schizosaccharomyces pombe* CDK 7,8,9 and the top 20 Hits have been extracted, each. Amino Acid Sequences were aligned with ClustalOmega 1.2.2. (Sievers and Higgins (2018) (https://doi.org/10.1002/pro.3290)) and a Neighbor joining consensus tree was calculated with the Jukes Cantor Genetic distance Model with 1000 bootstraps replicates. *Paramecium* kinases are highlighted according to the color-code for gene expression categories visualized by the scheme above.
